# (2*E*)-1-(3,5-Di­hydroxy­phen­yl)-3-(4-meth­oxy­phen­yl)prop-2-en-1-one

**DOI:** 10.1107/S1600536814009155

**Published:** 2014-04-26

**Authors:** K. S. Ezhilarasi, D. Reuben Jonathan, Shanmugam Sathya, K. Prathebha, G. Usha

**Affiliations:** a PG and Research Department of Physics, Queen Mary’s College, Chennai-4, Tamilnadu, India; b PG and Research Department of Chemistry, Presidency College, Chennai-5, Tamil Nadu, India

## Abstract

In the title compound, C_16_H_14_O_4_, the benzene rings are inclined to one another by 4.91 (7)°. The conformation about the C=O and C=C bonds is *trans* and *cis*, respectively. In the crystal, mol­ecules are linked by O—H⋯O hydrogen bonds, forming inversion dimers with an *R*_2_^2^(14) ring motif. The dimers are linked *via* O—H⋯O and C—H⋯O hydrogen bonds, forming undulating two-dimensional networks lying parallel to (10-1). These networks are linked by further C—H⋯O hydrogen bonds, forming a three-dimensional structure.

## Related literature   

For the biological activity of chalcone derivatives, see: Shenvi *et al.* (2013 ▶); Sharma *et al.* (2012 ▶); Hsieh *et al.* (2012 ▶); Sashidhara *et al.* (2011 ▶). For related structures, see: Ahn *et al.* (2013 ▶); Jasinski *et al.* (2011 ▶). For standard bend lengths, see: Allen *et al.* (1987 ▶). For the synthesis, see: Shettigar *et al.* (2006 ▶); Patil *et al.* (2007 ▶). For graph-set notation, see: Bernstein *et al.* (1995 ▶).
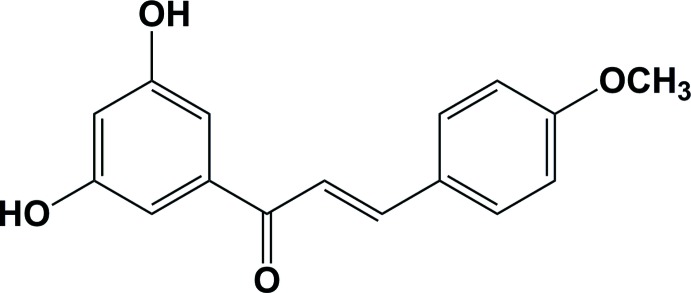


## Experimental   

### 

#### Crystal data  

C_16_H_14_O_4_
*M* *_r_* = 270.28
Monoclinic, 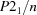

*a* = 9.1920 (9) Å
*b* = 13.8931 (13) Å
*c* = 10.9299 (10) Å
β = 106.619 (2)°
*V* = 1337.5 (2) Å^3^
*Z* = 4
Mo *K*α radiation
μ = 0.10 mm^−1^
*T* = 293 K
0.22 × 0.20 × 0.20 mm

#### Data collection  

Bruker Kappa APEXII CCD diffractometer
Absorption correction: multi-scan (*SADABS*; Bruker, 2004 ▶) *T*_min_ = 0.979, *T*_max_ = 0.981
13378 measured reflections
3402 independent reflections
2617 reflections with *I* > 2σ(*I*)
*R*_int_ = 0.032

#### Refinement  

*R*[*F* ^2^ > 2σ(*F* ^2^)] = 0.042
*wR*(*F* ^2^) = 0.137
*S* = 0.91
3384 reflections
181 parameters
H-atom parameters constrained
Δρ_max_ = 0.22 e Å^−3^
Δρ_min_ = −0.17 e Å^−3^

### 

Data collection: *APEX2* (Bruker, 2004 ▶); cell refinement: *APEX2* and *SAINT* (Bruker, 2004 ▶); data reduction: *SAINT* and *XPREP* (Bruker, 2004 ▶); program(s) used to solve structure: *SIR92* (Altomare *et al.*, 1993 ▶); program(s) used to refine structure: *SHELXL97* (Sheldrick, 2008 ▶); molecular graphics: *ORTEP-3 for Windows* (Farrugia, 2012 ▶); software used to prepare material for publication: *SHELXL97* and *PLATON* (Spek, 2009 ▶).

## Supplementary Material

Crystal structure: contains datablock(s) I, New_Global_Publ_Block. DOI: 10.1107/S1600536814009155/su2727sup1.cif

Structure factors: contains datablock(s) I. DOI: 10.1107/S1600536814009155/su2727Isup2.hkl

Supporting information file. DOI: 10.1107/S1600536814009155/su2727Isup3.cml

CCDC reference: 998945

Additional supporting information:  crystallographic information; 3D view; checkCIF report

## Figures and Tables

**Table 1:** Hydrogen-bond geometry (Å, °)

*D*—H⋯*A*	*D*—H	H⋯*A*	*D*⋯*A*	*D*—H⋯*A*
O2—H2⋯O1^i^	0.82	1.90	2.7196 (15)	174
O3—H3*A*⋯O2^ii^	0.82	2.03	2.8361 (14)	167
C3—H3⋯O2^ii^	0.93	2.59	3.2875 (17)	132
C5—H5⋯O1^i^	0.93	2.43	3.1498 (17)	134
C12—H12⋯O4^iii^	0.93	2.47	3.3926 (16)	169

Symmetry codes: (i) 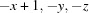; (ii) 
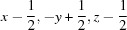
; (iii) 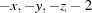.

**Fig. 1.**
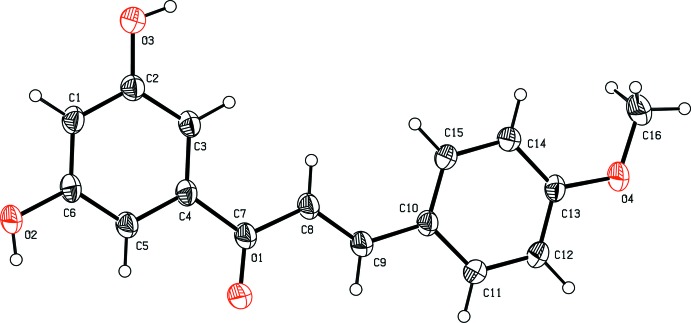
The molecular structure of the title molecule, with atom labelling. Displacement ellipsoids are drawn at the 30% probability level.

**Fig. 2.**
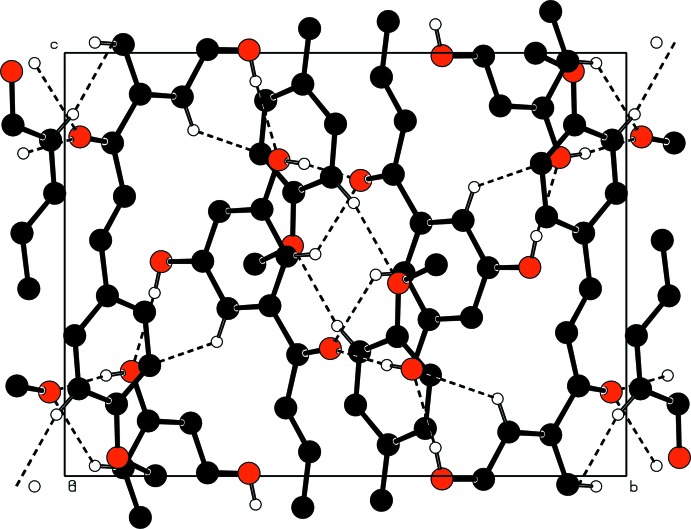
The crystal packing of the title compound viewed along the a axis. The dashed lines indicate the hydrogen bonds (see Table 1 for details).

## supplementary crystallographic information

### 1. Comment

Chalcones are one of the secondary metabolites in plants and belong to a class of flavonoid. They have shown diverse biological activities including anticancer (Shenvi *et al.*, 2013), antimicrobial (Sharma *et al.* 2012), antidiabetic (Hsieh *et al.*, 2012) and antiinflammatory (Sashidhara *et al.*, 2011). As part of our attempt to investigate how the substituent effects of chalcones effect the biological activities of various compounds, the title compound was synthesized and its crystal structure is reported herein.

The molecuar structure of the title compound is illustrated in Fig. 1. The bond lengths (Allen *et al.*, 1987) and bond angles are within normal values. The benzene rings (C1-C6 and C10-C15) are inclined to one another by 4.91 (7) °. The torsion angles about the C7═O1 and C8═C9 bonds confirm the *trans* and *cis* conformations of the respective bonds. The C8═ C9 bond distance is 1.332 (2) Å, and is in good agreement with the value [1.329 (3) Å] reported for a similar structure (Ahn *et al.*, 2013). The methoxy C atom and hydroxy O atoms are almost coplanar with the benezene ring to which they are attached. The bond angles C4—C7—C8 = 120.4 (1)° and C8—C9—C10 = 128.2 (1)° differ slightly from the normal values but are comparable with the values reported for a similar structure (Jasinski *et al.*, 2011).

In the crystal, molecules are linked by O—H···O hydrogen bonds forming inversion dimers with a graph set motif of *R*^2^_2_(14) [Bernstein *et al.*, 1995]. The dimers are linked by O-H···O and C-H···O hydrogen bonds forming undulating two-dimensional networks lying parallel to (10-1) [Fig. 2 and Table 1]. These networks are linked by further C-H···O hydrogen bonds forming a three-dimensional structure (Table 1).

### 2. Experimental

The title compound was syntheized by the base catalyzed Claisen-Schmidt reaction according to the published procedures (Shettigar *et al.*, 2006; Patil *et al.*, 2007). In a 250 ml round-bottomed flask 3,5-hydroxyacetophenone (0.05 mol) and 4-methoxybenzaldehyde (0.05 mol) were placed and 120 ml of absolute alcohol were added. The mixture was stirred at room temperature for 5 min. Then 20 ml of 20% sodium hydroxide solution was added and the mixture was stirred for 2 h. The precipitate generated by adding a sufficient amount of dilute hydrochloric acid was filtered, washed with water and dried. The crude product was recrystallized twice from absolute alcohol yielding colourless block-like crystals (Yield 79%; M.p. 477 K).

### 3. Refinement

H atoms were positioned geometrically and treated as riding atoms: O—H = 0.82 Å, C—H =0.93 – 0.96 Å, with *U*_iso_(H)= 1.5*U*_eq_(O and C-methyl) and = 1.2Ueq(C) for other H atom.

### Crystal data

**Table:**

C_16_H_14_O_4_	*F*(000) = 568
*M**_r_* = 270.28	*D*_x_ = 1.342 Mg m^−^^3^
Monoclinic, *P*2_1_/*n*	Mo *K*α radiation, λ = 0.71073 Å
Hall symbol: -P 2yn	Cell parameters from 3402 reflections
*a* = 9.1920 (9) Å	θ = 2.4–28.5°
*b* = 13.8931 (13) Å	µ = 0.10 mm^−^^1^
*c* = 10.9299 (10) Å	*T* = 293 K
β = 106.619 (2)°	Block, colourless
*V* = 1337.5 (2) Å^3^	0.22 × 0.20 × 0.20 mm
*Z* = 4	

### Data collection

**Table:**

Bruker Kappa APEXII CCD diffractometer	3402 independent reflections
Radiation source: fine-focus sealed tube	2617 reflections with *I* > 2σ(*I*)
Graphite monochromator	*R*_int_ = 0.032
ω and φ scan	θ_max_ = 28.5°, θ_min_ = 2.4°
Absorption correction: multi-scan (*SADABS*; Bruker, 2004)	*h* = −10→12
*T*_min_ = 0.979, *T*_max_ = 0.981	*k* = −18→18
13378 measured reflections	*l* = −14→14

### Refinement

**Table:**

Refinement on *F*^2^	Primary atom site location: structure-invariant direct methods
Least-squares matrix: full	Secondary atom site location: difference Fourier map
*R*[*F*^2^ > 2σ(*F*^2^)] = 0.042	Hydrogen site location: inferred from neighbouring sites
*wR*(*F*^2^) = 0.137	H-atom parameters constrained
*S* = 0.91	*w* = 1/[σ^2^(*F*_o_^2^) + (0.0868*P*)^2^ + 0.2822*P*] where *P* = (*F*_o_^2^ + 2*F*_c_^2^)/3
3384 reflections	(Δ/σ)_max_ < 0.001
181 parameters	Δρ_max_ = 0.22 e Å^−^^3^
0 restraints	Δρ_min_ = −0.17 e Å^−^^3^

### Special details

**Table:**

Geometry. All e.s.d.'s (except the e.s.d. in the dihedral angle between two l.s. planes) are estimated using the full covariance matrix. The cell e.s.d.'s are taken into account individually in the estimation of e.s.d.'s in distances, angles and torsion angles; correlations between e.s.d.'s in cell parameters are only used when they are defined by crystal symmetry. An approximate (isotropic) treatment of cell e.s.d.'s is used for estimating e.s.d.'s involving l.s. planes.
Refinement. Refinement of *F*^2^ against ALL reflections. The weighted *R*-factor *wR* and goodness of fit *S* are based on *F*^2^, conventional *R*-factors *R* are based on *F*, with *F* set to zero for negative *F*^2^. The threshold expression of *F*^2^ > σ(*F*^2^) is used only for calculating *R*-factors(gt) *etc*. and is not relevant to the choice of reflections for refinement. *R*-factors based on *F*^2^ are statistically about twice as large as those based on *F*, and *R*- factors based on ALL data will be even larger.

### Fractional atomic coordinates and isotropic or equivalent isotropic displacement parameters (Å^2^)

**Table:**

	*x*	*y*	*z*	*U*_iso_*/*U*_eq_	
C1	0.24996 (15)	0.22432 (10)	0.12529 (12)	0.0470 (3)	
H1	0.2457	0.2550	0.1998	0.056*	
C2	0.15728 (15)	0.25425 (10)	0.00768 (12)	0.0447 (3)	
C3	0.16469 (14)	0.20929 (10)	−0.10413 (11)	0.0429 (3)	
H3	0.1017	0.2292	−0.1827	0.051*	
C4	0.26647 (14)	0.13466 (9)	−0.09755 (11)	0.0404 (3)	
C5	0.35787 (15)	0.10425 (10)	0.01976 (11)	0.0431 (3)	
H5	0.4258	0.0538	0.0243	0.052*	
C6	0.34818 (14)	0.14885 (10)	0.13033 (11)	0.0427 (3)	
C7	0.28664 (15)	0.08504 (10)	−0.21300 (12)	0.0455 (3)	
C8	0.18574 (15)	0.10638 (10)	−0.33903 (11)	0.0440 (3)	
H8	0.1070	0.1502	−0.3473	0.053*	
C9	0.20462 (15)	0.06396 (10)	−0.44272 (12)	0.0456 (3)	
H9	0.2855	0.0212	−0.4289	0.055*	
C10	0.11515 (14)	0.07605 (9)	−0.57409 (11)	0.0414 (3)	
C11	0.14639 (15)	0.01784 (10)	−0.66808 (12)	0.0461 (3)	
H11	0.2250	−0.0267	−0.6448	0.055*	
C12	0.06338 (16)	0.02522 (10)	−0.79376 (12)	0.0474 (3)	
H12	0.0849	−0.0147	−0.8547	0.057*	
C13	−0.05233 (15)	0.09213 (10)	−0.82969 (11)	0.0423 (3)	
C14	−0.08502 (16)	0.15125 (11)	−0.73877 (12)	0.0477 (3)	
H14	−0.1623	0.1967	−0.7627	0.057*	
C15	−0.00197 (16)	0.14207 (10)	−0.61259 (12)	0.0474 (3)	
H15	−0.0252	0.1812	−0.5517	0.057*	
C16	−0.24683 (19)	0.16157 (14)	−0.99921 (14)	0.0642 (4)	
H16A	−0.2893	0.1554	−1.0899	0.096*	
H16B	−0.2083	0.2257	−0.9792	0.096*	
H16C	−0.3240	0.1492	−0.9578	0.096*	
O1	0.39025 (14)	0.02703 (10)	−0.19868 (9)	0.0712 (4)	
O2	0.43646 (11)	0.11864 (8)	0.24713 (8)	0.0539 (3)	
H2	0.4898	0.0736	0.2377	0.081*	
O3	0.06007 (13)	0.32813 (8)	0.00670 (10)	0.0626 (3)	
H3A	0.0110	0.3399	−0.0671	0.094*	
O4	−0.12717 (12)	0.09454 (8)	−0.95602 (9)	0.0559 (3)	

### Atomic displacement parameters (Å^2^)

**Table:**

	*U*^11^	*U*^22^	*U*^33^	*U*^12^	*U*^13^	*U*^23^
C1	0.0550 (7)	0.0512 (8)	0.0324 (6)	−0.0020 (6)	0.0088 (5)	−0.0046 (5)
C2	0.0490 (7)	0.0439 (7)	0.0394 (6)	−0.0002 (5)	0.0098 (5)	−0.0002 (5)
C3	0.0462 (6)	0.0460 (7)	0.0327 (6)	−0.0006 (5)	0.0052 (5)	0.0019 (5)
C4	0.0452 (6)	0.0422 (7)	0.0303 (5)	−0.0040 (5)	0.0051 (5)	−0.0014 (4)
C5	0.0463 (6)	0.0459 (7)	0.0321 (6)	0.0004 (5)	0.0033 (5)	−0.0018 (5)
C6	0.0462 (6)	0.0480 (7)	0.0290 (5)	−0.0075 (5)	0.0027 (5)	−0.0010 (5)
C7	0.0523 (7)	0.0479 (7)	0.0312 (6)	0.0038 (5)	0.0035 (5)	−0.0010 (5)
C8	0.0495 (7)	0.0465 (7)	0.0312 (6)	0.0022 (5)	0.0036 (5)	0.0003 (5)
C9	0.0508 (7)	0.0479 (7)	0.0336 (6)	0.0053 (5)	0.0048 (5)	−0.0008 (5)
C10	0.0479 (6)	0.0437 (7)	0.0302 (5)	0.0002 (5)	0.0073 (5)	−0.0022 (5)
C11	0.0519 (7)	0.0478 (7)	0.0378 (6)	0.0087 (6)	0.0116 (5)	−0.0020 (5)
C12	0.0592 (7)	0.0504 (8)	0.0337 (6)	0.0040 (6)	0.0149 (5)	−0.0068 (5)
C13	0.0503 (7)	0.0463 (7)	0.0282 (5)	−0.0033 (5)	0.0081 (5)	−0.0024 (5)
C14	0.0536 (7)	0.0487 (8)	0.0366 (6)	0.0107 (6)	0.0062 (5)	−0.0043 (5)
C15	0.0574 (8)	0.0496 (7)	0.0322 (6)	0.0086 (6)	0.0079 (5)	−0.0079 (5)
C16	0.0666 (10)	0.0746 (11)	0.0406 (7)	0.0090 (8)	−0.0018 (6)	0.0036 (7)
O1	0.0817 (8)	0.0862 (9)	0.0363 (5)	0.0398 (7)	0.0017 (5)	−0.0036 (5)
O2	0.0619 (6)	0.0627 (6)	0.0286 (4)	0.0080 (5)	−0.0006 (4)	−0.0031 (4)
O3	0.0747 (7)	0.0664 (7)	0.0434 (5)	0.0224 (5)	0.0116 (5)	−0.0024 (5)
O4	0.0678 (6)	0.0644 (7)	0.0293 (4)	0.0076 (5)	0.0036 (4)	−0.0044 (4)

### Geometric parameters (Å, º)

**Table:**

C1—C6	1.374 (2)	C10—C15	1.3850 (18)
C1—C2	1.3873 (18)	C10—C11	1.4002 (17)
C1—H1	0.9300	C11—C12	1.3712 (17)
C2—O3	1.3589 (16)	C11—H11	0.9300
C2—C3	1.3913 (18)	C12—C13	1.3823 (19)
C3—C4	1.3846 (18)	C12—H12	0.9300
C3—H3	0.9300	C13—O4	1.3555 (14)
C4—C5	1.3829 (16)	C13—C14	1.3868 (18)
C4—C7	1.4952 (18)	C14—C15	1.3780 (17)
C5—C6	1.3835 (17)	C14—H14	0.9300
C5—H5	0.9300	C15—H15	0.9300
C6—O2	1.3681 (14)	C16—O4	1.4152 (19)
C7—O1	1.2230 (16)	C16—H16A	0.9600
C7—C8	1.4542 (16)	C16—H16B	0.9600
C8—C9	1.3320 (18)	C16—H16C	0.9600
C8—H8	0.9300	O2—H2	0.8200
C9—C10	1.4460 (16)	O3—H3A	0.8200
C9—H9	0.9300		
			
C6—C1—C2	119.21 (12)	C15—C10—C11	117.67 (11)
C6—C1—H1	120.4	C15—C10—C9	123.40 (11)
C2—C1—H1	120.4	C11—C10—C9	118.93 (12)
O3—C2—C1	117.50 (12)	C12—C11—C10	121.31 (12)
O3—C2—C3	121.97 (12)	C12—C11—H11	119.3
C1—C2—C3	120.53 (12)	C10—C11—H11	119.3
C4—C3—C2	119.52 (11)	C11—C12—C13	119.83 (12)
C4—C3—H3	120.2	C11—C12—H12	120.1
C2—C3—H3	120.2	C13—C12—H12	120.1
C5—C4—C3	119.95 (12)	O4—C13—C12	115.54 (11)
C5—C4—C7	116.91 (12)	O4—C13—C14	124.36 (12)
C3—C4—C7	123.12 (11)	C12—C13—C14	120.10 (11)
C4—C5—C6	119.95 (12)	C15—C14—C13	119.39 (12)
C4—C5—H5	120.0	C15—C14—H14	120.3
C6—C5—H5	120.0	C13—C14—H14	120.3
O2—C6—C1	118.62 (11)	C14—C15—C10	121.69 (12)
O2—C6—C5	120.56 (12)	C14—C15—H15	119.2
C1—C6—C5	120.82 (11)	C10—C15—H15	119.2
O1—C7—C8	121.19 (12)	O4—C16—H16A	109.5
O1—C7—C4	118.41 (11)	O4—C16—H16B	109.5
C8—C7—C4	120.40 (12)	H16A—C16—H16B	109.5
C9—C8—C7	120.85 (12)	O4—C16—H16C	109.5
C9—C8—H8	119.6	H16A—C16—H16C	109.5
C7—C8—H8	119.6	H16B—C16—H16C	109.5
C8—C9—C10	128.17 (13)	C6—O2—H2	109.5
C8—C9—H9	115.9	C2—O3—H3A	109.5
C10—C9—H9	115.9	C13—O4—C16	118.40 (11)
			
C6—C1—C2—O3	179.03 (12)	C4—C7—C8—C9	178.67 (13)
C6—C1—C2—C3	−0.8 (2)	C7—C8—C9—C10	179.53 (13)
O3—C2—C3—C4	179.68 (12)	C8—C9—C10—C15	5.0 (2)
C1—C2—C3—C4	−0.6 (2)	C8—C9—C10—C11	−174.38 (14)
C2—C3—C4—C5	1.14 (19)	C15—C10—C11—C12	−0.5 (2)
C2—C3—C4—C7	−177.38 (12)	C9—C10—C11—C12	178.91 (13)
C3—C4—C5—C6	−0.4 (2)	C10—C11—C12—C13	0.9 (2)
C7—C4—C5—C6	178.19 (12)	C11—C12—C13—O4	179.54 (12)
C2—C1—C6—O2	−178.54 (12)	C11—C12—C13—C14	−0.4 (2)
C2—C1—C6—C5	1.5 (2)	O4—C13—C14—C15	179.57 (13)
C4—C5—C6—O2	179.12 (12)	C12—C13—C14—C15	−0.5 (2)
C4—C5—C6—C1	−0.9 (2)	C13—C14—C15—C10	0.9 (2)
C5—C4—C7—O1	−6.2 (2)	C11—C10—C15—C14	−0.4 (2)
C3—C4—C7—O1	172.31 (14)	C9—C10—C15—C14	−179.79 (14)
C5—C4—C7—C8	174.30 (12)	C12—C13—O4—C16	−179.90 (14)
C3—C4—C7—C8	−7.1 (2)	C14—C13—O4—C16	0.0 (2)
O1—C7—C8—C9	−0.8 (2)		

### Hydrogen-bond geometry (Å, º)

**Table:**

*D*—H···*A*	*D*—H	H···*A*	*D*···*A*	*D*—H···*A*
O2—H2···O1^i^	0.82	1.90	2.7196 (15)	174
O3—H3*A*···O2^ii^	0.82	2.03	2.8361 (14)	167
C3—H3···O2^ii^	0.93	2.59	3.2875 (17)	132
C5—H5···O1^i^	0.93	2.43	3.1498 (17)	134
C12—H12···O4^iii^	0.93	2.47	3.3926 (16)	169

Symmetry codes: (i) −*x*+1, −*y*, −*z*; (ii) *x*−1/2, −*y*+1/2, *z*−1/2; (iii) −*x*, −*y*, −*z*−2.
